# Effects of Aging on Motor Unit Properties in Isometric Elbow Flexion

**DOI:** 10.3390/bioengineering12080869

**Published:** 2025-08-12

**Authors:** Fang Qiu, Xiaodong Liu, Chen Chen

**Affiliations:** 1 School of Physical Education & Health, Shanghai University of International Business and Economics, Shanghai 201620, China; qiufang@suibe.edu.cn; 2 Institute of Physical Education, Guangxi University of Science and Technology, Liuzhou 545000, China; 3 State Key Laboratory of Mechanical System and Vibration, Shanghai Jiao Tong University, Shanghai 200240, China

**Keywords:** motor unit, aging, elbow flexion, discharge property, neural drive

## Abstract

This study investigates age-related differences in motor unit (MU) properties and neuromuscular control during isometric elbow flexion across the human lifespan. High-density surface electromyography (sEMG) was recorded from the biceps brachii of 44 participants, divided into three groups: Child (8–14 years), Adult (20–40 years), and Elder (65–80 years). MU spike trains were extracted noninvasively by sEMG decomposition. Then the discharge rate, MU action potential (MUAP) morphology, recruitment threshold, and common neural drive were quantified and compared across age groups. This study provides novel insights into force tracking performance, revealing that both children and elders exhibit higher errors compared to young adults, likely due to immature or declining motor control systems. Significant differences in MU discharge patterns were observed across force levels and age groups. Children and elders displayed lower MU discharge rates at low force levels, which increased at higher forces. In contrast, adults demonstrated higher MU action potential peak-to-peak amplitudes (PPV) and recruitment thresholds (RTs), along with steeper PPV-RT slopes, suggesting a narrower RT range in children and older adults. Principal component analysis revealed a strong correlation between common neural drive and force across all groups, with neural drive being weaker in elders. Overall, young adults exhibited the most efficient and synchronized MU control, while children and older adults showed distinct deviations in discharge intensity, recruitment strategies, and neural synergy. These findings comprehensively characterize MU adaptations across the lifespan, offering implications for developmental neurophysiology and age-specific neuromuscular diagnostics and interventions.

## 1. Introduction

Aging is associated with a progressive decline in neuromuscular function, manifesting in reduced muscle strength, impaired coordination, and slower movement execution [1,2]. These changes contribute significantly to functional limitations and increased risk of falls and disability in older adults [3,4]. Physically, such impairments result from both muscular and neural alterations, including sarcopenia, neuromuscular junction degeneration, and reduced central drive [5]. However, while the morphological and contractile consequences of aging in skeletal muscle have been extensively studied, the neural mechanisms underlying age-related changes in motor control remain less clearly defined [6]. In particular, how motor unit (MU) behavior adapts across the lifespan requires further investigation to understand the interplay between the central and peripheral nervous systems in shaping motor function [7,8].

Recent advances in high-density surface electromyography (sEMG) and signal decomposition techniques have opened new avenues for examining the neural control of movement noninvasively [9]. Blind source separation algorithms, such as convolution kernel compensation (CKC) and fast independent component analysis, allow for the accurate extraction of individual MU spike trains (MUSTs) and MU action potentials (MUAPs) from high-density sEMG recordings [10,11,12]. These methods have been validated in both healthy and clinical populations and enable the direct quantification of MU firing behavior, recruitment properties, and the structure of common neural inputs to motor neuron pools [13,14]. With these tools, researchers can now move beyond global sEMG metrics to examine the underlying neural mechanisms of force generation, providing an opportunity to clarify how aging modulates the architecture and function of the neuromuscular system [15,16].

A growing body of work has used intramuscular or sEMG decomposition to characterize age-related changes in MU behavior. In elderly adults, reductions in mean discharge rate and increased inter-spike interval have been observed during both low- and moderate-force contractions, indicating a decline in both excitatory input and motor neuron responsiveness [17,18]. Similarly, studies report increased recruitment thresholds and reduced MUAP amplitudes, likely reflecting a loss of high-threshold MUs and subsequent reinnervation by lower-threshold axons, resulting in fewer but larger MUs [19,20]. In children, the motor system is still undergoing maturation, characterized by reduced MU discharge rates, greater variability in firing patterns, and less differentiated recruitment hierarchies [21,22]. Adults, by contrast, exhibit the most stable discharge behavior, stronger common synaptic input to motor pools, and more efficient recruitment strategies, consistent with peak neuromuscular performance during early and middle adulthood [23]. These developmental and degenerative patterns highlight the dynamic nature of MU control across the lifespan.

Despite these insights, most studies have focused on specific age segments (e.g., only the elderly vs. young adults), which limits the ability to track age-related changes across the entire human lifespan. There is a lack of studies that directly compare MU behavior between children, adults, and elders using unified sEMG decomposition frameworks. In addition, few studies have examined how common neural drive, quantified through methods such as principal component analysis (PCA) or cumulative spike train (CST) analysis, varies across different age groups. Yet, common input is a key determinant of MU synchronization and functional force output [24,25].

The present study investigates MU properties across three age groups (children, adults, and elders) during isometric elbow flexion to address these gaps. Using a CKC-based sEMG decomposition algorithm, we extracted MU discharge features, MUAP morphology, recruitment thresholds, and common neural input during a force-tracking task at four contraction levels. By comparing these parameters across the lifespan, we aimed to identify key developmental and aging-related transitions in MU behavior and neuromuscular coordination.

The study offers several new contributions beyond previous research on age-related MU changes. First, it uses high-density sEMG and decomposition techniques to noninvasively analyze MU properties across the entire human lifespan—including Child, Adult, and Elder groups—which sets it apart methodologically from earlier studies focusing on specific age ranges. Another advancement is the combination of EMG decomposition with PCA to compare common neural drive and synergy patterns across age groups. Additionally, the study shows that children and elders depend on coarser neural strategies during low-force tasks, while adults exhibit more refined control. It also provides new insights into force tracking performance, revealing that both children and elders make more errors than adults, likely due to underdeveloped or declining motor control systems. These results highlight age-specific neuromuscular adaptations that were previously unquantified in such detail. In sum, this work offers a comprehensive, noninvasive characterization of age-related changes in MU control and sheds light on the neural mechanisms underlying motor decline and development.

## 2. Materials and Methods

### 2.1. Subjects

A total of 44 participants, ranging in age from 8 to 80 years, were recruited for the study. They were categorized into three groups based on age: the Child group (under 14 years, comprising 14 participants, with nine males), the Adult group (20 to 40 years, comprising 15 participants, with nine males), and the Elder group (over 65 years, comprising 15 participants, with eight males). The average ages for the three groups were 11 ± 1, 29 ± 7, and 73 ± 3 years, with corresponding average body mass indices of 16.8 ± 1.7, 25.9 ± 5.2, and 22.6 ± 2.2, respectively. We selected 40 as the upper limit for the Adult group because ages 20–40 typically represent the period of peak motor performance, where muscle strength, coordination, and neural plasticity are stable and less confounded by age-related decline (e.g., sarcopenia, slower reaction times) [8].

All participants had no neurological disorders, injuries, or operations related to upper limb movement, and provided informed consent before participating. All participants had no long-term sports training experience and completed the International Physical Activity Questionnaire before the experiment. According to the questionnaire results, the majority of participants fell into the moderate physical activity category, which means the subjects typically perform activities with a metabolic equivalent of task (MET) of 3–6, including brisk walking, cycling, dancing, golf, and similar activities. Notably, all participants in the elderly group were classified as having moderate activity levels. During participant screening, we required that subjects refrain from strenuous activity for 48 h before the experiment. The experimental protocol and informed consent process adhered to the Declaration of Helsinki and received approval from the local ethics committee of Shanghai Jiao Tong University (approval number E20240248I).

### 2.2. Experiments

#### 2.2.1. Force Signal Recording

**Figure 1:**
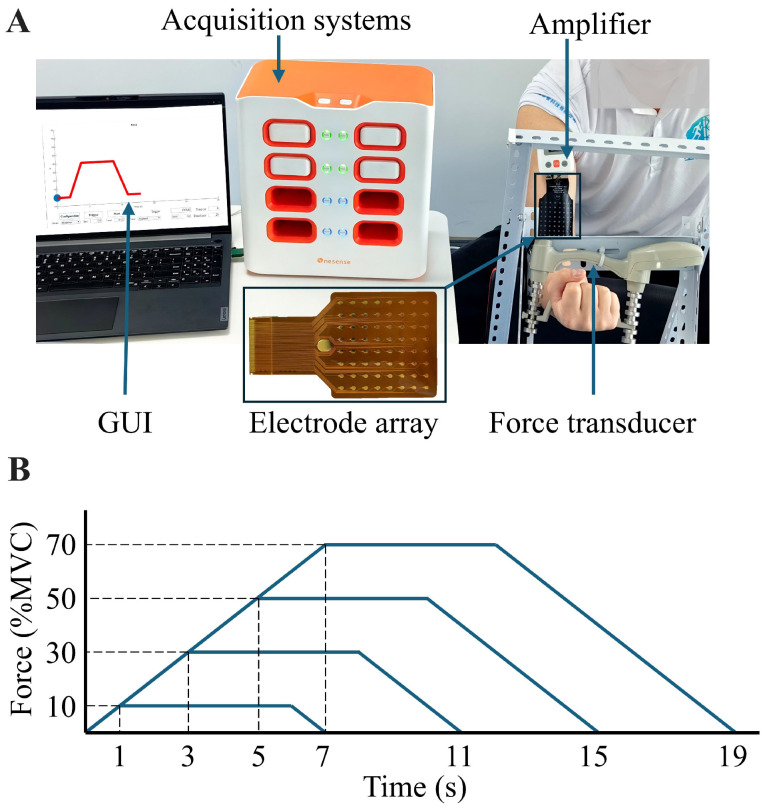
Experimental setup. (**A**) illustrates the hardware used to record sEMG and force. (**B**) shows the four force curves that the subjects were instructed to follow.

A custom-designed platform was constructed to capture force signals during elbow flexion tasks. A force sensor (Hand Grip Dynamometer, Biometrics Ltd., Newport, UK) was securely affixed to a stationary stand (Figure 1A). During the experimental procedures, participants were seated in a comfortable position with the force acquisition platform positioned directly in front of them. Participants extended their dominant arm, maintaining an approximate 100 ° angle between the upper arm and forearm, ensuring the midpoint of the forearm was in consistent contact with the force sensor. Only the dominant arm, used more frequently in daily life and training, was selected due to its stable movement performance. The non-dominant arm might exhibit greater variability and individual differences across subjects. To minimize extraneous movements, the participant’s elbow was stabilized using a strap, and participants were instructed to engage their upper arm muscles predominantly during force exertion.

A graphical user interface was developed using MATLAB to facilitate real-time data acquisition from the force sensor via a serial port and to provide visual feedback to the participants. The force sensor was configured to operate at a sampling frequency of 200 Hz.

#### 2.2.2. High-Density sEMG Recording

The high-density sEMG electrodes were employed to capture a broader range of muscle activities and decode MU discharges. SEMG signals were acquired using a 64-channel electrode array arranged in an 8 × 8 configuration (Grid8X8P-8mm, Onesense, Suzhou, China). Each electrode had a diameter of 3 mm, with an inter-electrode spacing of 8 mm in both directions (Figure 1A). The electrode array was positioned through palpation, ensuring that the array center was located over the belly of the biceps brachii muscle. The electrode array covers an area of about 60 mm × 60 mm and is sufficient to encompass both heads of the biceps brachii. The array was connected to a multichannel acquisition module, which transmitted the data via Wi-Fi to a base station (Oct-HD, Onesense, Suzhou, China) linked to a laptop.

The sEMG signals were recorded in monopolar mode with a gain of 1000 and a sampling rate of 2000 Hz. The sEMG signals were hardware bandpass filtered between 3 Hz and 900 Hz and digitized with a 24-bit analog-to-digital converter. The signals were displayed and recorded in real time using the Oct-HD PC Client software on the laptop and subsequently saved for offline analysis. During the experimental phase, we visually inspected the signal quality empirically and adjusted electrode placement, reference electrodes, or connectors until the signal quality met the standard. To ensure synchronization between the force signals and sEMG signals, a custom synchronization module based on an Arduino microcontroller was implemented. The microcontroller sent trigger signals to the sEMG acquisition base station at the start and end of force signal acquisition.

#### 2.2.3. Experimental Protocol

Participants were required to perform isometric elbow flexion tasks during the experiment. Before the experiment began, each participant underwent a maximum voluntary contraction (MVC) test. Specifically, participants were instructed to exert the maximum isometric contraction force of the elbow flexion three times, with a 1 min rest between each contraction. The participants were instructed to hold for more than 3 s each time. The maximum forces of three times were averaged as the MVC force.

During each task, participants were asked to control their contraction force to follow a trapezoidal curve, which included a ramp-up phase, a steady phase, and a ramp-down phase. The trapezoidal curve’s peak contraction force levels included four intensities: 10%, 30%, 50%, and 70% of the MVC. For each intensity level, the rate of force increase and decrease remained constant (10%MVC/s). After reaching the target force level, participants were required to maintain the force steadily for 5 s before decreasing it at the same rate to return to a relaxed state. Figure 1B illustrates the trapezoidal curves corresponding to different force paradigms. For each force intensity level, participants performed two repeated trials. For each subject, the order of the four force-level tasks was randomized. Throughout the experiment, a screen in front of the participants displayed the target force curve and the real-time contraction force they generated.

### 2.3. Data Analysis

**Figure 2:**
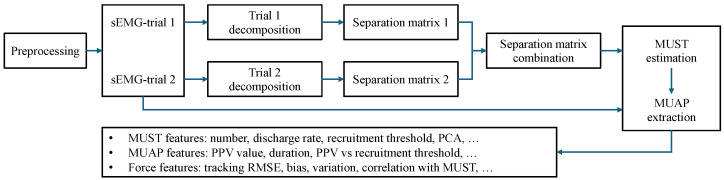
Diagram of data analysis. After preprocessing, the sEMG signals from two repetitive trials were first decomposed individually and then combined for MUST estimation and MUAP extraction. Several features related to MUSTs, MUAPs, and force tracking were extracted for the following analysis.

The data analysis workflow is illustrated in Figure 2. After preprocessing, the sEMG signals were decomposed to extract MUSTs. Features related to the intrinsic properties of MUs, such as MUAPs and discharge rate, as well as force-related features, including tracking accuracy and recruitment thresholds, were subsequently extracted.

#### 2.3.1. Preprocessing

For the sEMG signals, a 4th-order Butterworth filter was applied for bandpass filtering between 20 Hz and 500 Hz, complemented by a 50 Hz comb filter to eliminate low-frequency motion artifact and powerline interference. The root mean square (RMS) value was calculated for each channel. If a channel’s RMS deviates by more than five standard deviations from the mean, that channel is discarded. Typically, fewer than five channels are removed, ensuring no significant impact on subsequent EMG decomposition. For the force signals, the data were first up-sampled to 2000 Hz, followed by low-pass filtering at 2.5 Hz to attenuate noise.

#### 2.3.2. sEMG Decomposition

The blind source separation algorithm based on CKC was employed to decompose the sEMG signals [10]. In brief, the sEMG signals were treated as a multi-input multi-output system, modeled as the convolutional mixture of MUSTs and MUAPs [26]. In the CKC algorithm, by extending the sEMG signals, this process was simplified into an instantaneous mixing model. CKC extracts the discharge pulses of MUs by solving the Linear Minimum Mean Square Error (LMMSE) estimation of MUSTs [27].

(1) $$\hat{\theta }=C_{\theta x}^{T}C_{xx}^{-1}x$$

where $\theta =E\left(x\theta^{T}\right)={\left[\theta_{1}\left(n\right),\theta_{1}\left(n-1\right),\ldots ,\theta_{1}\left(n-L+1\right),\ldots ,\theta_{J}\left(n\right),\ldots ,\theta_{J}\left(n-L+1\right)\right]}^{T}$ is the extended version of all pulse trains, $C_{\theta x}=\left[c_{\theta_{1}x},c_{\theta_{2}x},\ldots ,c_{\theta_{J}x}\right]$ contains the cross-correlation vector between each pulse train and sEMG signals (*x*), $C_{xx}=E\left(xx^{T}\right)$ is the covariance matrix of sEMG signals, *E* denotes the mathematical expectation.

In this study, a natural gradient descent algorithm was used to iteratively estimate the cross-correlation vector ( $c_{\theta_{j}x}$ ) and extract MUSTs [28]. Each task included two repetitive trials. We first decomposed the sEMG signals from each trial, then the separation matrix ( $C_{\theta x}^{T}C_{xx}^{-1}$ ) of each trial was reserved to decompose the other trials. The MUSTs identified from the separation matrices of the two trials were merged and filtered. A signal-based measurement, pulse-to-noise ratio (PNR), was used to evaluate the decomposition accuracy [29]. MUSTs with a PNR below 25 dB or a discharge frequency exceeding 35 Hz were excluded.

The multichannel MUAP waveforms of each MU were obtained by spike-triggered averaging [30]. For each channel, signals from a 64 ms interval, which were triggered by the discharge timings of each MU, were collected and averaged.

#### 2.3.3. Feature Extraction

For the force signals, three metrics, root mean square error (RMSE), Bias error, and Coefficient of Variation (CoV), were extracted to assess the force tracking performance across different age groups [31,32]. The definitions of these metrics are provided below.

(2) $$RMSE=\sqrt{\frac{1}{N}\sum_{i}^{N}{\left(F_{Mi}-F_{Di}\right)}^{2}}$$

(3) $$Bias\;error=\frac{1}{N}\sum_{i}^{N}\left(F_{Mi}-F_{Di}\right)$$

(4) $$CoV_{force}=\frac{std(detrend\left(F_{M}\right))}{mean(F_{M})}$$

where $F_{Mi}$ is the measured contraction force at sample *i*, $F_{Di}$ is the corresponding desired force, *N* is the number of samples. The $F_{M}$ in Equation (4) indicates the measured force in the steady phase. The best straight-fit line was removed from $F_{M}$ when calculating the standard deviation.

For the characterization of MU discharge properties, the mean discharge rate during the steady-state phase of isometric contraction was extracted [33]. For the MUSTs, only discharge pulses occurring within the steady-force phase were retained. The instantaneous discharge rate of MUs was derived by applying a 400 ms Hanning window to the MUSTs for moving average computation, followed by averaging the discharge rate curve over the steady-state phase.

For the characterization of MUAPs, the maximum peak-to-peak value (PPV) across all channels of the MUAP waveform was extracted, along with the duration of the PPV for the corresponding channel. The duration was defined as the temporal interval between the maximum and minimum values within the MUAP waveform.

By integrating the force signals and MUSTs, we further extracted the recruitment threshold (RT) for each MU, defined as the contraction force at the time of the MU’s first discharge [6,34]. Additionally, for the MUs decoded from each task, a linear regression analysis was performed using the least squares method to fit the relationship between RT and MUAP PPV. The slope and intercept of the linear equation were retained for subsequent analysis. The fitting errors exhibited greater variability when there were few MUs with RT distributed in a low range. Therefore, only the tasks where the RT range was higher than 30%MVC were included for linear fitting.

Finally, we analyzed the common drive information of MUs. For each trial’s MUST signals, the discharge rate curves of each MU were first computed, followed by PCA. The number of variables corresponded to the number of MUSTs, and the sample size was equal to the number of sampling points in a trial. We calculated the variance explained by the first principal component (PC1) in the feature space and the Pearson correlation coefficient between PC1 and the contraction force curve. To evaluate the relationship between the cumulative spike train and the PC1, the Pearson correlation coefficient between these two measures was also computed [35].

### 2.4. Statistics

A one-way analysis of variance was applied to evaluate the effect of age on the features described above. The factor (independent variable) was age groups (Child, Adult, Elder), and the dependent variables were features. The homogeneity of variance for the variables was first tested. If satisfied, the Bonferroni method was conducted. If not, Dunnett’s C method was used instead. The significance level was set to 0.05. The symbols ∗, ∗∗ , and ∗∗∗ denote the significance level of 0.01<p<0.05 , 0.001≤p≤0.01 , and p<0.001 , respectively. Only the significance level is illustrated in the manuscript, while the detailed statistical analysis results, including overall between-group effect size (Eta-squared) and confidence intervals, are given in the supplementary data.

All the decomposition and analyses were implemented in MATLAB 2024a (MATLAB Inc., Natick, MA, USA).

## 3. Results

### 3.1. Motor Unit Decomposition Across Ages

**Figure 3:**
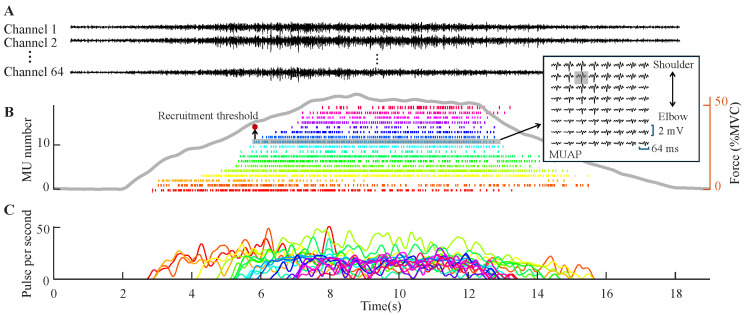
Example decomposition results. (**A**) shows example sEMG signals from 3 of 64 channels in a trial of 50%MVC contraction. The corresponding MUSTs decomposed from sEMG are given in (**B**). Each vertical bar indicates a discharge of one MU. The discharge trains of each MU are denoted with different colors. The gray line is the measured force in this trial. An example of multichannel MUAP waveforms is shown in the subgraph, where the gray box indicates the channel with the maximum PPV. (**C**) shows the discharge rate curves of each MU corresponding to the MUSTs in (**B**).

Figure 3 illustrates a representative example of sEMG decomposition at 50%MVC from an adult participant. The MUSTs and discharge rates reveal temporally structured discharge patterns from 18 MUs, with each discharge synchronized to the generated force curve. Table 1 summarizes decomposition metrics across age groups and contraction intensities. The Adult group consistently exhibited the highest number of decomposed MUs at low forces, with an average of 19 ± 5 MUs at 10%MVC. The Child and Elder groups showed 15 ± 6 and 13 ± 6 MUs, respectively. At higher forces (70%MVC), MU numbers declined in all groups but remained relatively balanced: Child (8 ± 6), Adult (10 ± 4), Elder (11 ± 5). The PNR values remained stable and high across all groups and intensities (ranging from 29.1 ± 2.8 dB to 30.2 ± 3.1 dB), indicating consistently high decomposition quality.

### 3.2. Force Tracking Performance

**Figure 4:**
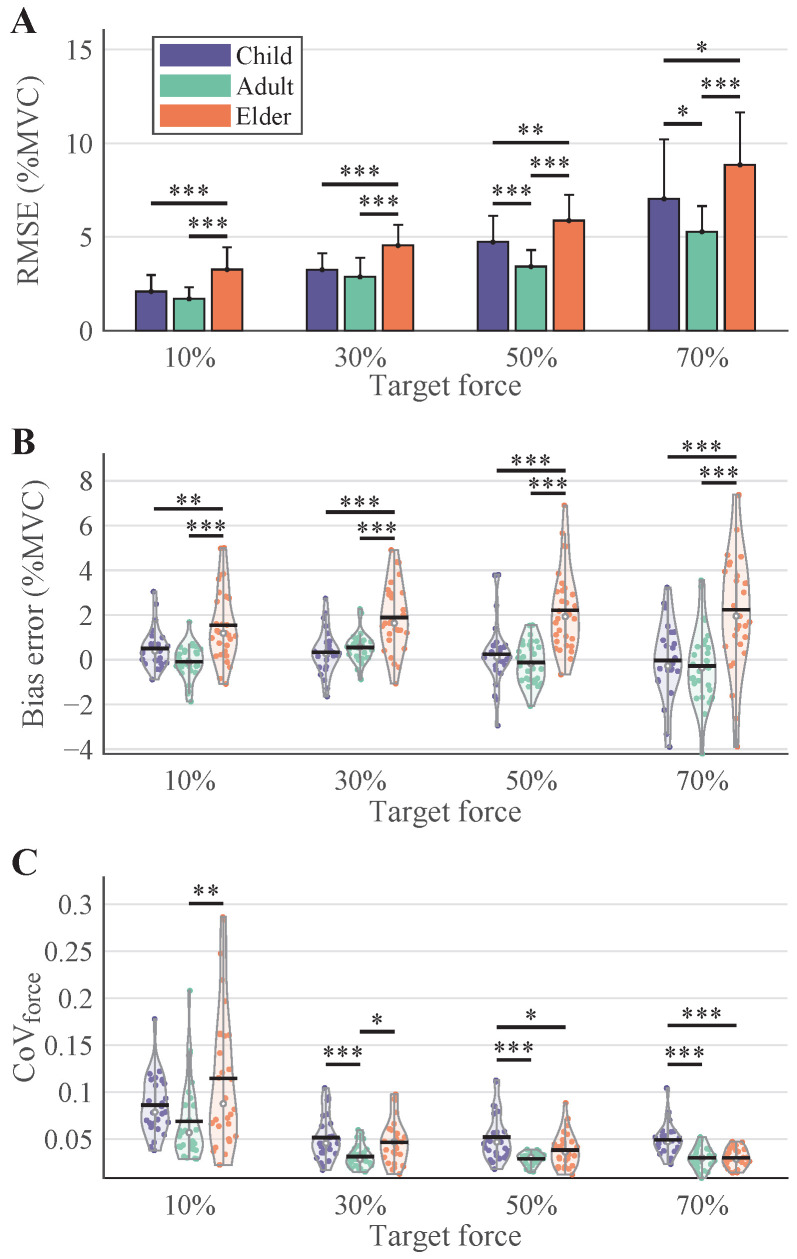
Force tracking performance across age groups. (**A**–**C**) illustrate the RMSE, Bias error, and $CoV_{force}$ , respectively. The results of each age group are indicated with colors.

Force control metrics are presented in Figure 4. The Adult group achieved the best force tracking performance with significantly lower RMSEs across all contraction levels. At 10%, 30%, 50%, and 70%MVC, RMSEs in adults were 1.70% ± 0.62%, 2.87% ± 1.00%, 3.42% ± 0.88%, and 5.28% ± 1.37%MVC, respectively. In contrast, the Elder group showed RMSEs of 3.26% ± 1.17%, 4.54% ± 1.09%, 5.87% ± 1.38%, and 8.85% ± 2.78%MVC at the same force levels (*p* < 0.001). The Child group exhibited similar impairments as the Elder group, but no significant difference from the Adult group at 10% and 30%MVC.

Similar trends were observed for the bias error (Figure 4B) and the coefficient of variation of force (Figure 4C). At different force levels, the Bias error of the Elder group was consistently significantly higher than that of the other two groups. As for $CoV_{force}$ , the Adult group consistently exhibited the lowest variability. Overall, $CoV_{force}$ tended to decrease at higher force levels, with a more pronounced reduction observed in the Elder group. At 10% and 30%MVC, the $CoV_{force}$ of the Elder group was significantly higher than that of the Adult group. However, no significant difference was observed between these two groups at higher force levels. At this point, the $CoV_{force}$ of the Child group became significantly higher than those of the other two groups.

### 3.3. Motor Unit Discharge and MUAP Morphology Properties

**Figure 5:**
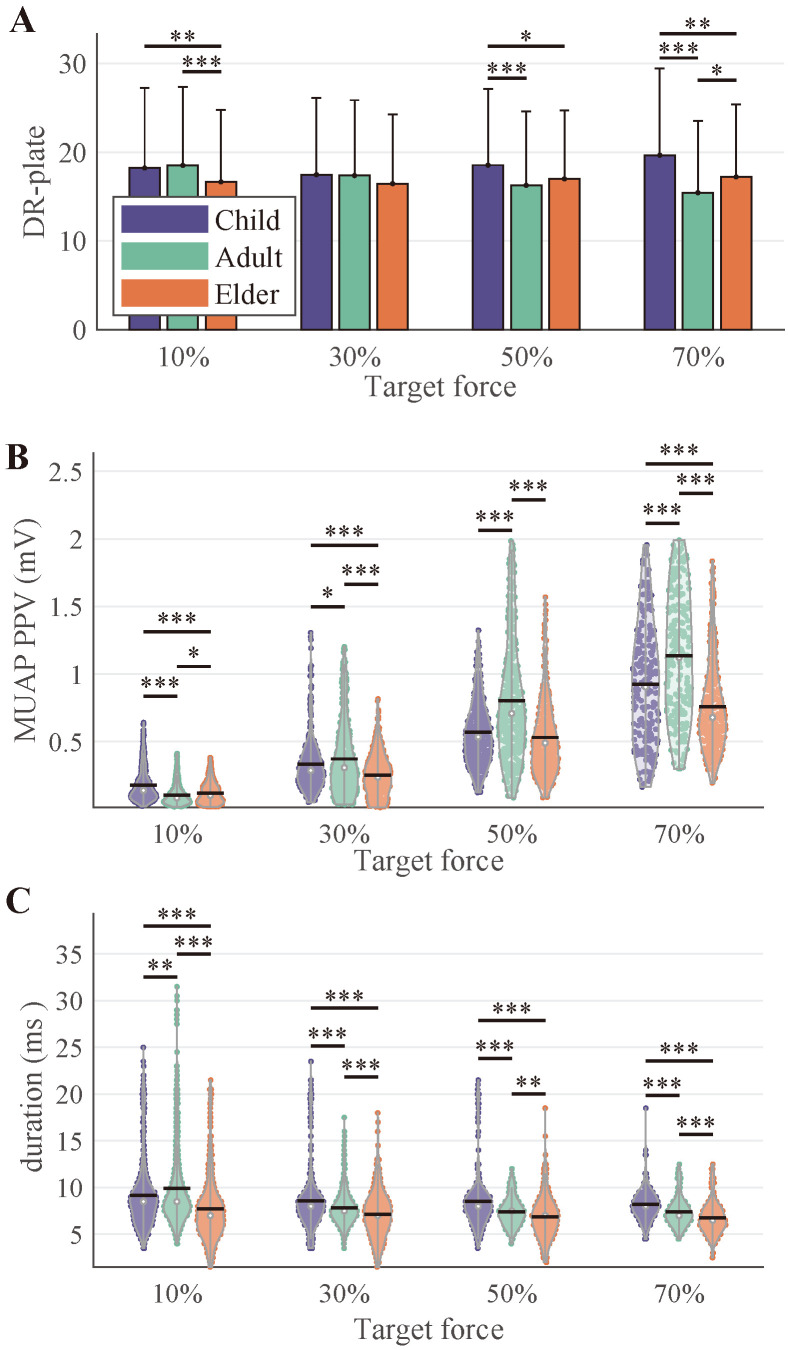
Discharge properties across different age groups. (**A**) illustrates the average discharge rate in the steady phase, respectively. pps: pulse per second. (**B**,**C**) give the MUAP PPV and corresponding duration, respectively.

Figure 5 summarizes the discharge behavior and MUAP morphological properties. At 10%MVC, the Elder group exhibited the lowest mean discharge rate, which was significantly lower than that of the other two groups. At 30%MVC, no significant differences were observed among the three groups. At higher force levels (50% and 70%MVC), the Child group showed significantly higher mean discharge rates compared to the other groups. With increasing force levels, the trends in mean discharge rate varied across groups; the Child group showed a slight increase, the Adult group exhibited a decreasing trend, while the Elder group remained relatively stable.

MUAP morphology exhibited notable age effects. The MUAP PPV (Figure 5B) increased with contraction level and was significantly higher in adults across all force intensities. In contrast, both the Child and Elder groups displayed reduced PPVs, suggesting smaller and/or more spatially dispersed muscle fiber populations within MUs. Except at 10%MVC, aging was associated with a decrease in MUAP duration, with the Elder group exhibiting the shortest durations and the Child group the longest. However, at 10%MVC, the Adult group showed the longest MUAP duration among the three groups.

**Figure 6:**
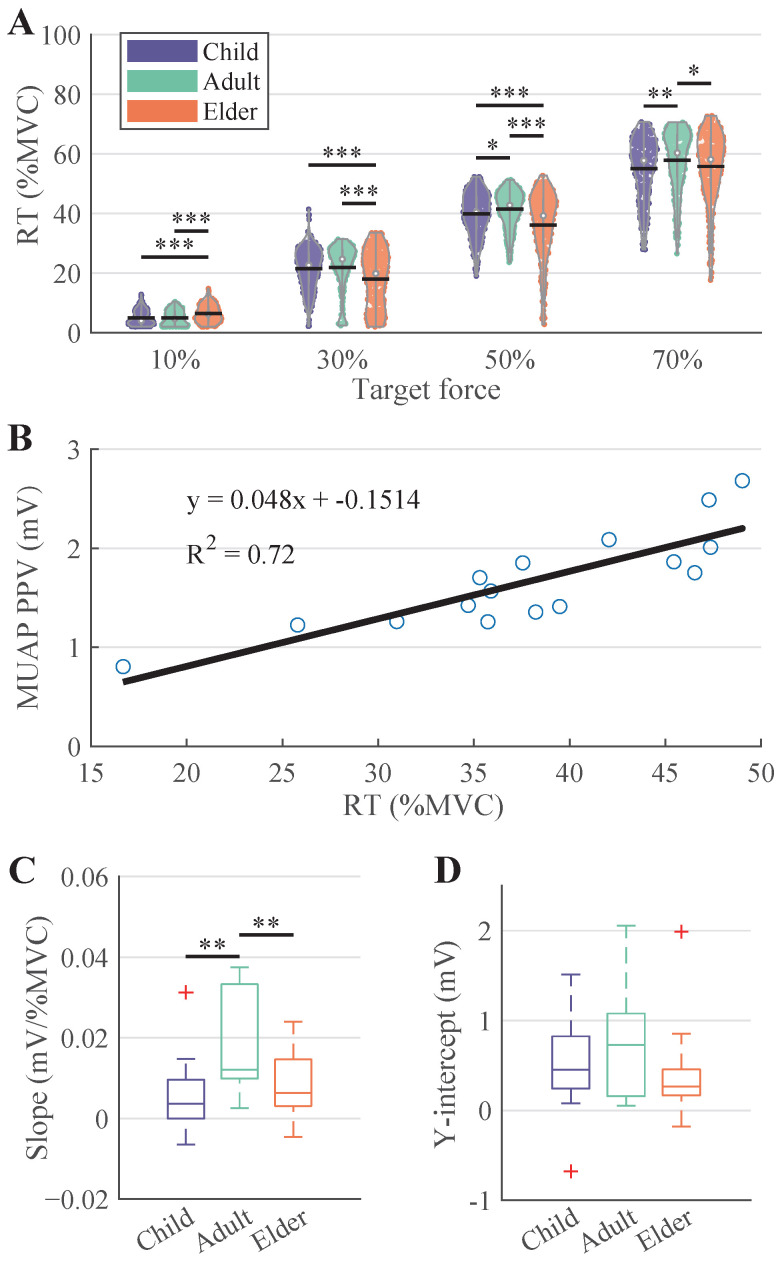
Relationship between recruitment thresholds and MUAP PPV. (**A**) illustrates the recruitment threshold results in each age group and contraction level. (**B**) shows an example linear fitting between MUAP PPV and RT from a subject in the Adult group. (**C**,**D**) give the slopes and Y-intercept values of all subjects in each age group.

Figure 6A shows that MU recruitment thresholds increased with force level and were consistently lower in the Elder group, especially at 50% and 70%MVC. A linear relationship between recruitment threshold and MUAP PPV was observed within subjects (Figure 6B). The regression slopes (Figure 6C) were highest in the Adult group, indicating a steeper association between recruitment force and MUAP amplitude. As to the y-intercepts (Figure 6D), there was no significant difference observed between the three age groups.

### 3.4. Common Neural Drive from MUSTs

**Figure 7:**
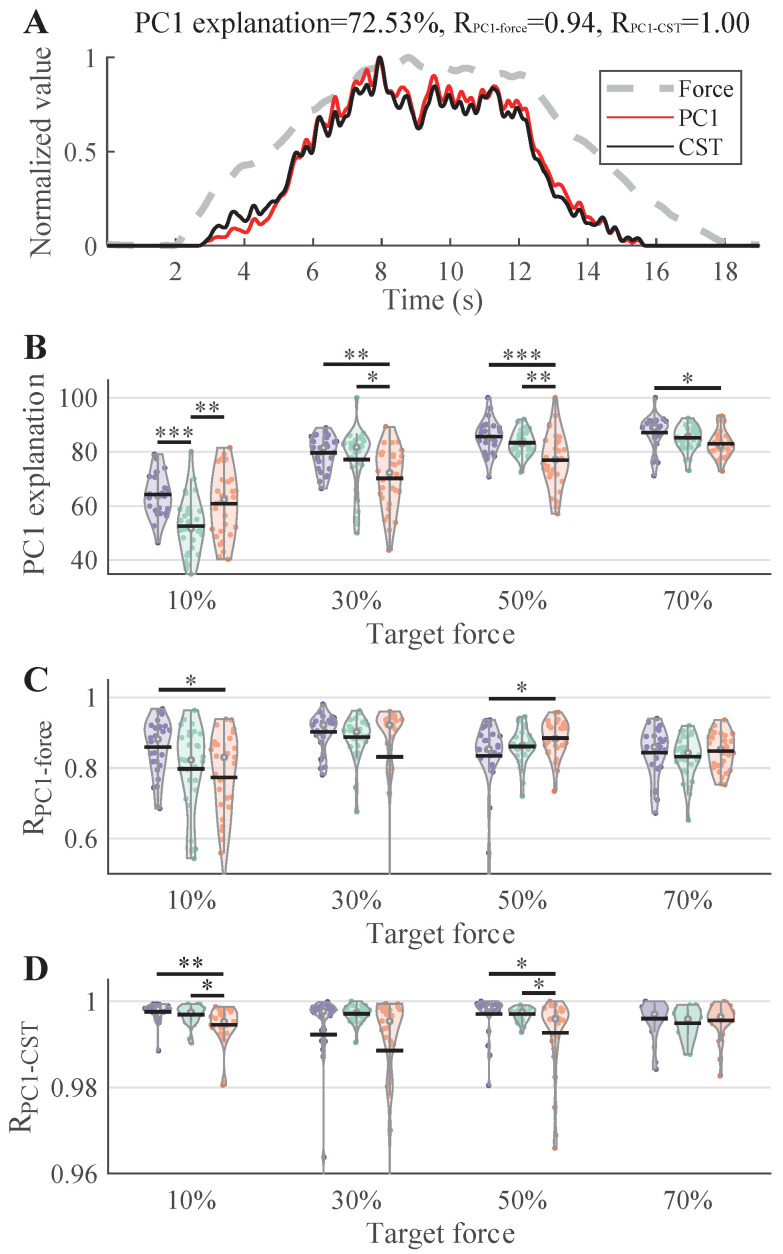
PCA and cumulative spike train results. (**A**) illustrates example PC1, discharge rate of cumulative spike train, and force signals in a trial of 50%MVC. (**B**–**D**) show the variance explanation of PC1, the correlation coefficients between PC1 and force, and the correlation between PC1 and cumulative spike train discharge rate, respectively.

Figure 7 illustrates the results from PCA of MU discharge rates. In a representative example (Figure 7A), the first principal component captured over 70% of the variance and was highly correlated with both the force trace and the cumulative spike train. Except at 10%MVC, the Elder group consistently showed the lowest variance explained by the first principal component. At 10%MVC, however, the Adult group exhibited a significantly lower variance explanation compared to the other two groups. Regarding the correlations between the first principal component and force, as well as the first principal component and cumulative spike train, all three groups demonstrated strong correlations; the average correlation between the first principal component and force exceeded 0.8, while the correlation between the first principal component and cumulative spike train was close to 1. In most cases, there were no significant differences among the three groups.

## 4. Discussion

### 4.1. Decomposition Performance Across Age Groups

This study confirms the effectiveness of CKC-based sEMG decomposition for characterizing MU activity across developmental and aging stages. Despite physiological and anatomical differences across age groups, the decomposition yielded consistently high pulse-to-noise ratios (PNR > 29 dB) and stable MU yields, demonstrating the robustness of the method. These results suggest that noninvasive decomposition techniques can be reliably applied across the lifespan, supporting their broader use in developmental and geriatric neuromuscular assessments. It is noteworthy that, despite differences in the exertion levels among participants across different groups, the number of decoded MUs remained relatively consistent. This is attributed to the inherent characteristics of the decomposition algorithm. Due to local convergence, existing decomposition algorithms often struggle to identify all active MU discharge activities. It is essential to note that the subsequent analyses in this study are based solely on the MUs that were successfully decoded.

In this study, the minimum duration for sEMG decomposition was 7 s for 10%MVC tasks, which is shorter than the decomposition durations used in most studies. However, previous research has demonstrated that the CKC algorithm can be effectively applied to even shorter sEMG segments (under 5 s) [36]. The decoding performance observed in this study also demonstrated the reliability of the decomposition. It should be noted that the analytical method employed in this study is not limited to a specific type of participant and can be fully applied to habitual exercisers, such as athletes. As concluded in many studies, individuals accustomed to regular exercise (e.g., athletes) exhibit significant differences in certain motor unit characteristics compared to untrained participants [34,37]. All subjects in this study had no long-term training or professional sports experience. If athletes had been included, the conclusions might have differed.

### 4.2. Age-Related Modulation of Force Generation and MU Properties

The impact of age on force generation was pronounced. As illustrated in Figure 4, in most cases, the RMSE and Bias error of force tracking were significantly higher in the Elder group compared to the other two groups. Aging tends to impair the stability of force maintenance, leading to increased tracking errors. However, it is noteworthy that the Child group also exhibited significantly higher force tracking errors than the Adult group, which may be attributed to the incomplete development of the motor control system in children. In the $CoV_{force}$ analysis, children also demonstrated higher variance. Interestingly, as the contraction level increased, the $CoV_{force}$ decreased significantly across all three groups, with the most pronounced reduction observed in the elderly. At higher contraction levels (50% and 70%MVC), there was no significant difference between the Adult and Elder groups. It should be noted that the order of the four force-level tasks was randomized in this study. Therefore, the analytical results presented should not be affected by task sequence effects.

It should be noted that the trapezoidal curve with linearly increasing force level was employed in this study, while the task complexity may not be linear. The effect of fatigue, motivation, or learning (especially in children and the elderly) could be a confounding factor. During the experiment, we provided participants with adequate rest periods between tasks to prevent fatigue. Additionally, before the experiment, participants underwent a training session until they fully understood and mastered the force exertion technique. Through these measures, we minimized potential nonlinear influencing factors as much as possible.

In low-force level tasks, the discharge frequency of MUs recruited by elderly participants was significantly lower than that of younger participants (Figure 5). In general, aging is associated with a reduction in muscle size and volume, primarily due to the loss of motor neurons and muscle fibers [38]. Notably, the preferential loss of type II muscle fibers shifts the contribution toward type I MUs, which typically exhibit lower discharge rates [19,39]. In addition, the contraction speed of MUs decreases with aging, leading to prolonged inter-spike intervals and reduced discharge rates [8]. Previous studies have shown that during early childhood, the proportion of type I muscle fibers is typically higher, which may account for the lower discharge rates observed in the pediatric group [40]. As the force level increased, the average discharge frequency in the Child and Elder group gradually rose, eventually matching that of the Adult group (at 30% and 50%MVC) and even becoming significantly higher (at 70%MVC). At higher force levels, elderly individuals may require stronger neural input to compensate for muscle strength decline, resulting in higher MU discharge frequencies.

The MUAP morphology further revealed clear distinctions between groups. Adults exhibited larger MUAP amplitudes, suggestive of compact MU territories and synchronized muscle fiber activation. These features are hallmarks of efficient neuromuscular transmission and have been associated with greater functional capacity [19]. The duration of MUAPs in elders typically tends to increase, which is associated with reduced nerve conduction velocity and the loss of fast-twitch fibers. However, in this study, elders exhibited shorter MUAP durations. Following motor neuron loss, surviving neurons may reinnervate denervated muscle fibers through collateral sprouting. These newly formed nerve terminals can temporarily demonstrate more synchronized firing, resulting in steeper MUAP rise and fall phases, thereby shortening the overall duration. Additionally, most of the older adults tested in this study maintained a moderate level of physical activity, which may help preserve better motor unit synchronization and faster contraction properties. As a result, their MUAP durations were closer to those of younger individuals or even shorter.

The recruitment threshold analyses provide further insight into MU control strategies. RTs increased with force across all groups. However, no consistent or significant trends were observed in the recruitment thresholds across the three groups. The recruitment thresholds exhibited a strong correlation with the MUAP PPV. The steeper linear relationship between recruitment threshold and MUAP amplitude in adults supports clearer size-based recruitment consistent with Henneman’s size principle [41]. In contrast, the flatter slopes in the Child and Elder groups may reflect underdeveloped or reorganized recruitment hierarchies, respectively.

Throughout development, both the structural and contractile properties of muscle undergo dynamic changes [40,42]. In contrast, aging is known to modify the mechanical properties of muscle tissues, including increased passive stiffness, decreased muscle compliance, and shifts in muscle fiber type composition [38]. Such changes can alter the responsiveness of muscle spindles and Golgi tendon organs, thereby affecting the processes of force production and transmission [43]. Consequently, the synchrony and recruitment patterns of MU discharges may also be affected [15,38]. To compensate for these age-related mechanical alterations, the central nervous system modulates discharge rates to maintain stable force output.

The findings in this study reflect combined data from both male and female participants. While previous research has identified sex-related differences in MU discharge and contractile properties [44,45], this study mainly focused on how aging affects MU characteristics. Initial analyses showed sex-based differences in certain parameters, including force-tracking error, MU discharge rates, MUAP amplitudes, and recruitment thresholds. Still, the overall patterns regarding the effects of age and contraction level on MU behavior were consistent across sexes. As a result, the data are presented together to provide a thorough view of how aging influences MU function.

### 4.3. Common Neural Drive and Synergy

PCA of MU discharge rates revealed age-specific patterns in the organization of common neural input. As the force level increased, the variance explained by the first principal component rose across all groups, indicating that the common drive input to the muscles became stronger during higher-force exertions. In most cases, the Elder group exhibited a lower variance explained by the first principal component, suggesting less synergistic and task-relevant neural modulation [23,24]. Interestingly, the adult group exhibited the lowest PC1 variance explanation at 10%MVC, suggesting that lower-effort tasks may be governed by more differentiated or distributed motor control strategies. In contrast, the higher PC1 variance observed in children and elders under the same condition could reflect a reliance on coarser neural strategies during underdeveloped or degraded control states [18]. In addition, the potential factors resulting in higher PC1 variance might be the differences in task familiarity (e.g., children’s inexperience with precise force control) and intrinsic motor variability (e.g., age-related noise in neural drive). The Elder group’s higher PC1 variance could also reflect compensatory strategies with reduced selective motor unit recruitment due to sarcopenia or neural degradation. Nonetheless, the strong correlations between the first principal component and both force output and cumulative spike train across all groups indicate that, despite age-related differences in synergy, the essential neural mechanisms underlying force generation remain preserved to a substantial degree. These findings also suggest that while neuromuscular strategies evolve over the lifespan, the fundamental structure of common synaptic input to MUs remains a conserved feature of human motor control.

### 4.4. Applications and Limitations

This study advances our understanding of how aging impacts motor performance and motor unit characteristics while providing neurophysiological evidence for related research. Therefore, the study offers valuable clinical diagnostic references, particularly for assessing abnormal muscle activity patterns (such as pathological changes or functional decline) from a motor unit perspective.

Although the present study provides a comprehensive cross-sectional analysis of MU properties across age groups, several limitations warrant consideration. The focus on isometric elbow flexion may not capture the complexity of neural strategies in dynamic or multijoint movements [46]. Prior work has shown that MU recruitment and discharge properties are task-dependent [17]. Future studies should incorporate varied dynamic or functional tasks (e.g., sit-to-stand, walking) to validate these findings in more ecologically relevant contexts. Furthermore, sEMG decomposition may underrepresent deep or small MUs, especially in elderly participants with changes in mechanical properties, muscle atrophy, or increased subcutaneous tissue. Longitudinal studies are needed to map developmental trajectories and aging-induced changes in MU behavior. Lastly, integrating cortical and spinal recordings with sEMG-based decomposition could provide more mechanistic insight into the descending and segmental contributions to age-related changes in motor control.

## 5. Conclusions

This study investigates MU discharge behavior, MUAP morphology, and common neural input across three age groups during controlled isometric contractions. Using sEMG decomposition combined with PCA, we found that young adults show the most stable, efficient, and synchronized neuromuscular control. In contrast, children and older adults have lower MU discharge rates at low force levels, which increase at higher forces. Young adults also display higher MUAP PPV and recruitment thresholds, with steeper PPV-RT slopes, indicating a narrower RT range in children and older adults. Principal component analysis confirms a strong correlation between common neural drive and force across all groups, although neural drive is reduced in older adults. These findings enhance our understanding of neuromuscular adaptations throughout the lifespan and highlight the potential of sEMG-derived biomarkers for developmental studies, aging research, and personalized rehabilitation.

## Figures and Tables

**Table 1:** Decomposition results of age groups and contraction intensities.

	Target Force	MVC Force (kg)	MU Number	PNR (dB)
Child	10%	7.8 ± 2.2	15 ± 6	29.8 ± 3.0
	30%		10 ± 5	29.8 ± 3.4
	50%		11 ± 6	29.7 ± 3.3
	70%		8 ± 6	29.8 ± 3.4
Adult	10%	21.8 ± 7.8	19 ± 5	29.3 ± 2.8
	30%		11 ± 6	29.5 ± 2.7
	50%		12 ± 5	30.2 ± 3.1
	70%		10 ± 4	29.9 ± 2.8
Elder	10%	14.6 ± 5.1	13 ± 6	29.1 ± 2.8
	30%		11 ± 5	29.4 ± 2.8
	50%		10 ± 5	29.9 ± 2.8
	70%		11 ± 5	30.0 ± 3.0

## Data Availability

The raw data supporting the conclusions of this article will be made available by the authors on request.

## Supplementary Materials

The following supporting information can be downloaded at: https://www.mdpi.com/article/10.3390/bioengineering12080869/s1, Table S1. Detail statistical results for Figure 4. For each sub-graph, the Eta-squared indicates the overall between-group effect size. The difference between various groups and the 95% confidence interval are shown in the following rows. Table S2. Detail statistical results for Figure 5. For each sub-graph, the Eta-squared indicates the overall between-group effect size. The difference between various groups and the 95% confidence interval are shown in the following rows. Table S3. Detail statistical results for Figure 6. For each sub-graph, the Eta-squared indicates the overall between-group effect size. The difference between various groups and the 95% confidence interval are shown in the following rows. Table S4. Detail statistical results for Figure 7. For each sub-graph, the Eta-squared indicates the overall between-group effect size. The difference between various groups and the 95% confidence interval are shown in the following rows.
