# Supplementary material for: Effects of Aging on Motor Unit Properties in Isometric Elbow Flexion
Source: Bioengineering (Basel). 2025 Aug 12;12(8):869. doi: 10.3390/bioengineering12080869 (PMC12383959; doi:10.3390/bioengineering12080869)
Supplement: Supplementary file 1 [file bioengineering-12-00869-s001.zip › bioengineering-3758227-supplementary.pdf]

Article

Supplementary data for: Effects of aging on motor unit properties in isometric elbow flexion

Fang Qiu [1], Xiaodong Liu [2,*] and Chen Chen [3,*]

[1] School of Physical Education & Health, Shanghai University of International Buisiness and Economics, Shanghai, China; qiufang@suibe.edu.cn
[2] Institute of Physical Education, Guangxi University of Science and Technology, Liuzhou, China; xiaodongliu_2010@163.com
[3] State Key Laboratory of Mechanical System and Vibration, Shanghai Jiao Tong University, Shanghai, China; cedric_chen@sjtu.edu.cn
* Correspondence: cedric_chen@sjtu.edu.cn (C.C.); xiaodongliu_2010@163.com (X.L.)

Table S1. Detail statistical results for Figure 4. For each sub-graph, the Eta-squared indicates the overall between-group effect size. The difference between various groups and the 95% confidence interval are shown in the following rows.

		Target force			
		10%	30%	50%	70%
Figure 4A	Eta-squared	0.350	0.352	0.414	0.270
	Child-Adult	0.389, [-0.196,0.974]	0.378, [-0.254,1.010]	1.308, [0.528,2.089]	1.756, [0.094,3.417]
	Child-Elder	-1.174, [-1.759,-0.589]	-1.294, [-1.931,-0.657]	-1.142, [-1.923,-0.362]	-1.815, [-3.463,-0.166]
	Adult-Elder	-1.563, [-2.143,-0.983]	-1.672, [-2.292,-1.052]	-2.451, [-3.210,-1.691]	-3.570, [-5.138,-2.003]
Figure 4B	Eta-squared	0.270	0.292	0.356	0.246
	Child-Adult	0.596, [-0.128,1.319]	-0.210, [-0.908,0.489]	0.369, [-0.520,1.258]	0.244, [-1.116,1.603]
	Child-Elder	-1.031, [-1.755,-0.308]	-1.553, [-2.252,-0.855]	-1.970, [-2.858,-1.081]	-2.268, [-3.618,-0.919]
	Adult-Elder	-1.627, [-2.331,-0.923]	-1.344, [-2.030,-0.658]	-2.339, [-3.212,-1.465]	-2.512, [-3.795,-1.229]
Figure 4C	Eta-squared	0.120	0.160	0.226	0.325
	Child-Adult	0.017, [-0.016,0.051]	0.020, [0.008,0.033]	0.023, [0.012,0.034]	0.019, [0.011,0.028]
	Child-Elder	-0.028, [-0.062,0.005]	0.005, [-0.008,0.018]	0.014, [0.003,0.025]	0.019, [0.011,0.028]
	Adult-Elder	-0.046, [-0.078,-0.013]	-0.015, [-0.028,-0.003]	-0.009, [-0.020,0.002]	0.000, [-0.008,0.008]

Table S2. Detail statistical results for Figure 5. For each sub-graph, the Eta-squared indicates the overall between-group effect size. The difference between various groups and the 95% confidence interval are shown in the following rows.

		Target force			
		10%	30%	50%	70%
Figure 5A	Eta-squared	0.008	0.003	0.014	0.027
	Child-Adult	-0.276, [-1.334,0.782]	0.098, [-1.278,1.475]	2.277, [0.910,3.644]	3.707, [1.855,5.558]
	Child-Elder	1.576, [0.402,2.751]	1.011, [-0.376,2.399]	1.549, [0.099,2.999]	2.400, [0.646,4.154]
	Adult-Elder	1.852, [0.766,2.939]	0.913, [-0.423,2.250]	-0.728, [-2.130,0.674]	-1.307, [-2.987,0.374]
Figure 5B	Eta-squared	0.101	0.051	0.136	0.175
	Child-Adult	0.071, [0.060,0.083]	-0.001, [-0.042,0.040]	-0.349, [-0.422,-0.276]	-0.478, [-0.612,-0.344]
	Child-Elder	0.059, [0.046,0.071]	0.121, [0.079,0.162]	0.022, [-0.054,0.099]	0.191, [0.065,0.318]
	Adult-Elder	-0.013, [-0.024,-0.001]	0.121, [0.082,0.161]	0.371, [0.297,0.446]	0.669, [0.549,0.790]
Figure 5C	Eta-squared	0.049	0.045	0.079	0.108
	Child-Adult	-0.736, [-1.219,-0.253]	0.746, [0.31,1.183]	1.143, [0.758,1.529]	0.859, [0.52,1.198]
	Child-Elder	1.443, [0.909,1.977]	1.407, [0.970,1.844]	1.649, [1.243,2.054]	1.360, [1.040,1.680]
	Adult-Elder	2.179, [1.685,2.673]	0.661, [0.237,1.084]	0.505, [0.112,0.899]	0.501, [0.195,0.806]

Table S3. Detail statistical results for Figure 6. For each sub-graph, the Eta-squared indicates the overall between-group effect size. The difference between various groups and the 95% confidence interval are shown in the following rows.

		Target force			
		10%	30%	50%	70%
Figure 6A	Eta-squared	0.059	0.042	0.067	0.012
	Child-Adult	0.057, [-0.424,0.538]	-0.353, [-1.772,1.065]	-1.713, [-3.105,-0.321]	-2.915, [-5.201,-0.630]
	Child-Elder	-1.421, [-1.902,-0.940]	3.492, [2.050,4.934]	3.697, [2.234,5.160]	-0.778, [-3.003,1.447]
	Adult-Elder	-1.478, [-2.022,-0.934]	3.845, [2.400,5.29]	5.410, [3.983,6.837]	2.137, [0.093,4.181]
Figure 6C	Eta-squared	0.241			
	Child-Adult	-0.013, [-0.023,-0.004]			
	Child-Elder	-0.002, [-0.009, 0.006]			
	Adult-Elder	0.012, [0.004, 0.020]			
Figure 6D	Eta-squared	0.105			
	Child-Adult	-0.360, [-0.956,0.236]			
	Child-Elder	0.129, [-0.364,0.622]			
	Adult-Elder	0.489, [-0.004,0.982]			

Table S4. Detail statistical results for Figure 7. For each sub-graph, the Eta-squared indicates the overall between-group effect size. The difference between various groups and the 95% confidence interval are shown in the following rows.

		Target force			
		10%	30%	50%	70%
Figure 7B	Eta-squared	0.186	0.141	0.208	0.092
	Child-Adult	11.666, [5.147,18.184]	2.532, [-3.821,8.884]	2.248, [-2.355,6.850]	1.893, [-1.569,5.354]
	Child-Elder	3.458, [-3.061,9.976]	9.447, [3.094,15.800]	8.654, [4.052,13.257]	4.117, [0.656,7.579]
	Adult-Elder	-8.208, [-14.613,-1.803]	6.916, [0.732,13.099]	6.406, [1.927,10.886]	2.225, [-1.039,5.488]
Figure 7C	Eta-squared	0.086	0.051	0.077	0.013
	Child-Adult	0.062, [-0.013,0.136]	0.015, [-0.070,0.100]	-0.027, [-0.072,0.019]	0.011, [-0.028,0.051]
	Child-Elder	0.086, [0.011,0.161]	0.071, [-0.014,0.156]	-0.051, [-0.097,-0.005]	-0.004, [-0.044,0.036]
	Adult-Elder	0.024, [-0.049,0.098]	0.056, [-0.028,0.139]	-0.024, [-0.068,0.021]	-0.015, [-0.053,0.022]
Figure 7D	Eta-squared	0.153	0.062	0.118	0.013
	Child-Adult	0.001, [-0.001,0.003]	-0.005, [-0.014,0.004]	0.000, [-0.004,0.004]	0.001, [-0.001,0.004]
	Child-Elder	0.003, [0.001,0.005]	0.004, [-0.005,0.013]	0.004, [0.001,0.008]	0.000, [-0.002,0.003]
	Adult-Elder	0.002, [0.000,0.004]	0.009, [0.000,0.017]	0.004, [0.001,0.008]	-0.001, [-0.003,0.002]
